# Transcriptomic and metabolomic analysis of Yukon *Thellungiella* plants grown in cabinets and their natural habitat show phenotypic plasticity

**DOI:** 10.1186/1471-2229-12-175

**Published:** 2012-10-01

**Authors:** David R Guevara, Marc J Champigny, Ashley Tattersall, Jeff Dedrick, Chui E Wong, Yong Li, Aurelie Labbe, Chien-Lu Ping, Yanxiang Wang, Paulo Nuin, G Brian Golding, Brian E McCarry, Peter S Summers, Barbara A Moffatt, Elizabeth A Weretilnyk

**Affiliations:** 1 Department of Biology, McMaster University, 1280 Main St. West, Hamilton, ON, L8S 4K1, Canada; 2 Department of Chemistry and Chemical Biology, McMaster University, 1280 Main St. West, Hamilton, ON, L8S 4K1, Canada; 3 Department of Biology, University of Waterloo, 200 University Ave. West, Waterloo, ON, N2L 3G1, Canada; 4 Palmer Research, Agricultural and Forestry Research Station, University of Alaska-Fairbanks, 533 East Fireweed Ave., Palmer, AK, 99645, USA; 5 Present address: Department of Molecular and Cellular Biology, University of Guelph, Guelph, ON, N1G 2W1, Canada; 6 Present address: Melbourne School of Land and Environment, University of Melbourne, Parkville, VIC, 3010, Australia; 7 Present address: Département de mathématiques et de statistique, Pavillon Alexandre-Vachon Université Laval, Québec, G1K 7P4, Canada

## Abstract

**Background:**

*Thellungiella salsuginea* is an important model plant due to its natural tolerance to abiotic stresses including salt, cold, and water deficits. Microarray and metabolite profiling have shown that *Thellungiella* undergoes stress-responsive changes in transcript and organic solute abundance when grown under controlled environmental conditions. However, few reports assess the capacity of plants to display stress-responsive traits in natural habitats where concurrent stresses are the norm.

**Results:**

To determine whether stress-responsive changes observed in cabinet-grown plants are recapitulated in the field, we analyzed leaf transcript and metabolic profiles of *Thellungiella* growing in its native Yukon habitat during two years of contrasting meteorological conditions. We found 673 genes showing differential expression between field and unstressed, chamber-grown plants. There were comparatively few overlaps between genes expressed under field and cabinet treatment-specific conditions. Only 20 of 99 drought-responsive genes were expressed both in the field during a year of low precipitation and in plants subjected to drought treatments in cabinets. There was also a general pattern of lower abundance among metabolites found in field plants relative to control or stress-treated plants in growth cabinets. Nutrient availability may explain some of the observed differences. For example, proline accumulated to high levels in cold and salt-stressed cabinet-grown plants but proline content was, by comparison, negligible in plants at a saline Yukon field site. We show that proline accumulated in a stress-responsive manner in *Thellungiella* plants salinized in growth cabinets and in salt-stressed seedlings when nitrogen was provided at 1.0 mM. In seedlings grown on 0.1 mM nitrogen medium, the proline content was low while carbohydrates increased. The relatively higher content of sugar-like compounds in field plants and seedlings on low nitrogen media suggests that *Thellungiella* shows metabolic plasticity in response to environmental stress and that resource availability can influence the expression of stress tolerance traits under field conditions.

**Conclusion:**

Comparisons between *Thellungiella* plants responding to stress in cabinets and in their natural habitats showed differences but also overlap between transcript and metabolite profiles. The traits in common offer potential targets for improving crops that must respond appropriately to multiple, concurrent stresses.

## Background

Abiotic stresses such as drought, sub-optimal or extreme temperatures and high salinity decrease plant growth and productivity and are responsible for major losses in crop yield [1]. Incentives for improving abiotic stress tolerance of crops has taken on renewed significance in the face of looming global food shortages that are anticipated consequences of climate change [2]. Plants that grow in high stress habitats have adaptations that can guide the development of more stress tolerant crops.

Plants can exploit different life strategies to cope with their environment [3]. Specialists may show strong local adaptation to very specific environments while generalists may acquire a more “all purpose” phenotype that allows for plant growth under a broader range of conditions. A third strategy has been described as the adaptive plastic response where a plant with a given genotype can display variable phenotypes that are environment dependent but ultimately contribute to improved fitness [4]. Plasticity is adaptive when the phenotype induced in each environment results in greater fitness than would the alternative phenotype [5]. One difficulty in evaluating the extent to which plasticity plays a role in stress tolerance is that stresses imposed in the laboratory fall short of replicating the complex natural environments that induce adaptive gene by environment interactions [3].

The application of carefully prescribed treatments under controlled laboratory conditions in growth cabinets has successfully identified stress responses and genes whose products are involved in stress tolerance (reviewed in [6]). However, to date few benefits predicted from tolerance traits identified using plants subjected to stress in laboratories have translated directly to improved crop performance under actual field conditions [7]. This gap reflects the difficulty of faithfully reproducing the complexities of a natural environment in a growth cabinet [3]. For example, the majority of QTLs for flowering time identified in growth cabinets are not the same as the ones identified in the field under natural conditions [8,9].

The importance of phenotyping complex adaptive traits in an ecologically relevant context is highlighted by research on the genetic regulation of the transition to flowering in plants. These “flowering time” genes have largely been discovered using Arabidopsis growing in climate-controlled environments and are responsive to cues such as variation in photoperiod or light quality, ambient temperature, plant growth, and vernalization (reviewed in [10]). QTL mapping for date of bolting in chamber conditions as well as at field sites in Rhode Island and North Carolina showed that only a single QTL was common to all environments, several QTL affected flowering time only in controlled environments and a number of novel QTL determined bolting date in the field [8]. More recently, genome-wide association analyses with multiple Arabidopsis ecotypes concluded similarly that most genes associated with flowering time under climate-controlled conditions were not significant for plants in a field plot in the north of France [11]. This outcome underscores the value of validating the role of stress-responsive genes and their products through comparisons involving field and cabinet conditions. With respect to gene expression, studies that have used microarray profiling to compare transcript profiles in plants under controlled conditions to those in field plants show that there are similarities but also major differences among the products found between these sources, underscoring the challenge of selecting suitable genes for crop improvement [12,13].

The crucifer *Thellungiella salsuginea* (also described as *Thellungiella halophila* and *Eutrema salsugineum*), herein referred to as *Thellungiella*, is found on highly saline soil in the Yukon Territory of Canada, a semi-arid, subarctic region. *Thellungiella* exhibits a significant innate capacity to tolerate water deficits, freezing temperatures, and high salinity [14,15]. Research on *Thellungiella* has benefited from its close phylogenetic relationship to *Arabidopsis thaliana*[16] and it shares many properties of this genetic model plant including small stature, prolific seed habit, capacity for genetic transformation, a relatively short life cycle of six to eight weeks and a compact, fully sequenced, diploid genome (http://www.phytozome.net/thellungiella.php, [17]). The combination of extreme native environment, high stress tolerance and genetic attributes makes *Thellungiella* an excellent genetic and physiological model for comparative studies involving field and cabinet grown plants.

Changes in gene expression and metabolite content have been reported in *Thellungiella* subjected to specific stresses under controlled environment conditions. Using a genotype of *Thellungiella* originating from China (Shandong Province), salt stress treatments in growth cabinets led to the up-regulation of only six genes compared to 40 genes in Arabidopsis plants [18]. Several salt-responsive genes were notable due to their higher constitutive expression in unstressed *Thellungiella*[18] compared to Arabidopsis. This observation suggests that a number of genes associated with salt exposure are constitutively expressed in *Thellungiella,* resulting in a plant with heightened natural resilience toward salt stress. Gene expression patterns have also been reported for the Yukon *Thellungiella* ecotype subjected to cold, drought or salinity treatments in controlled environment cabinets [19]. Exposure to drought stress resulted in the differential expression of 101 genes, compared to cold stress (76 genes) or salt stress (22 genes). Interestingly, only three genes showed increased expression with exposure to each of the three stresses studied leading to the conclusion that *Thellungiella* has rather specific responses to these stresses [19]. Metabolite profiling of Shandong *Thellungiella* responding to osmotic stresses including desiccation and salinity [20,21] offers a means to link the expression of stress-responsive genes to metabolic phenotypes. For example, Shandong *Thellungiella* undergoing salinity stress under controlled environment conditions was shown to accumulate higher levels of proline and sugar alcohols than unsalinized controls and this outcome was associated with the enhanced expression of transcripts encoding enzymes involved in their synthesis [22].

In this study we focused upon *Thellungiella* growing under native Yukon field conditions where multiple, concurrent stresses are the norm. We compared gene expression and metabolite profiles of field plants with those subjected to prescribed stress treatments in cabinets [19]. In addition to differences in morphology between plants found in the field and those grown in cabinets, we discovered that gene expression and metabolites associated with stress tolerance in growth cabinets are only partially recapitulated under field conditions in the Canadian Yukon. In particular, while Yukon *Thellungiella* plants salt-stressed in growth cabinets produced and accumulated proline in a stress-responsive manner, the proline content in leaves of field plants found growing on highly saline soil was, by comparison, low. Using seedlings grown on defined media we show that nitrogen availability can influence the array of organic solutes accumulated by Yukon *Thellungiella* under osmotic stress. The natural plasticity displayed by *Thellungiella* from morphology to chemical composition makes this plant a valuable model to test the functional significance of adaptive traits under stress including that associated with proline accumulation.

## Results and discussion

In this study we used the crucifer *Thellungiella salsuginea*, which is native to the semi-arid, subarctic region of Canada, to study gene products expressed under natural conditions and compared them with those identified in plants subjected to regimes of simulated stress in growth cabinets. Given the extreme environmental conditions present at our Yukon field site compared to tightly controlled conditions in our growth cabinets, we fully expected to find differences that were location-specific. However, we also anticipated that stress-responsive gene products essential for survival under salt, cold, and/or drought conditions should be expressed in plants regardless of whether the plants were grown and stressed in the field or in cabinets.

### Yukon field site conditions

The season for plant growth in the Yukon Territory of Canada is typically short and for *Thellungiella* this extends from early May to late July (Bruce Bennett, personal communication). In 2002 we developed sampling and shipping protocols suitable for RNA and metabolite extractions after collecting specimens from remote field locations. We then collected plants at a field site located approximately 40 km NW of Whitehorse returning to the same site over 1- to 2-week periods in early July 2003 and late June 2005.

At the field site *Thellungiella* plants grow on soil encrusted with salt deposits. Soil at the site is composed of an upper, mineral-organic layer approximately 18 cm deep that overlays a clay horizon. Chemical analysis of the upper layer (Additional file 1: Table S1) shows the soil to be highly alkaline with a pH of 8.3 and saline (the average soil electrical conductivity for the upper 20 cm is 15.7 dS m^-1^ or roughly equivalent to 160 mM NaCl). This soil is high in magnesium, sodium and sulfates, a feature shared by other saline soils in the area [23]. With respect to other nutrients, the soil has adequate levels of potassium and phosphorus (236 and 26 mg kg^-1^, respectively), whereas the total nitrogen content is low (0.26%; [24]). The Ca/Mg ratio is less than 1.0, characteristic for a serpentine soil while a typical soil usually has a Ca/Mg ratio exceeding 1.0 (reviewed in [25]).

For the Whitehorse, Yukon area, the average temperature for the growing season in 2003 and 2005 was approximately 20°C (Additional file 1: Table S1). In 2003, average high and low temperatures for the ten-day period prior to harvest were 22°C and 15°C, respectively. At the field site on the day of harvest, soil temperature was 17°C, the air temperature was 24°C, and the light intensity was 1500 *μ*moles m^–2^ s^–1^. In 2005, the average high and low temperatures for the ten-day period before harvest were 18°C and 7°C, respectively. At the field where plants were collected in 2005, the soil temperature was 18°C, the daytime air temperature was 24°C, and the light intensity was 1433 *μ*moles m^–2^ s^–1^.

The average cumulative precipitation for this region is 86.9 mm from May through July, the growing season for *Thellungiella*, classifying this area as semi-arid (Environment Canada climate normals 1971 – 2000; http://www.climate.weatheroffice.gc.ca/climate_normals/index_e.html). In 2003, however, total precipitation prior to harvest was 50% lower than the long-term norms and, as such, can be considered a drought year even for a semi-arid region (Additional file 1: Figure S1; [26]). In contrast, precipitation prior to the 2005 harvest was 2.4-fold greater than the long-term norms. Thus prevailing levels of light and temperature during harvest for the two years were comparable but precipitation was very low in 2003 relative to 2005.

### *Thellungiella* plants in the field and in growth chambers have different phenotypes

**Figure 1:**
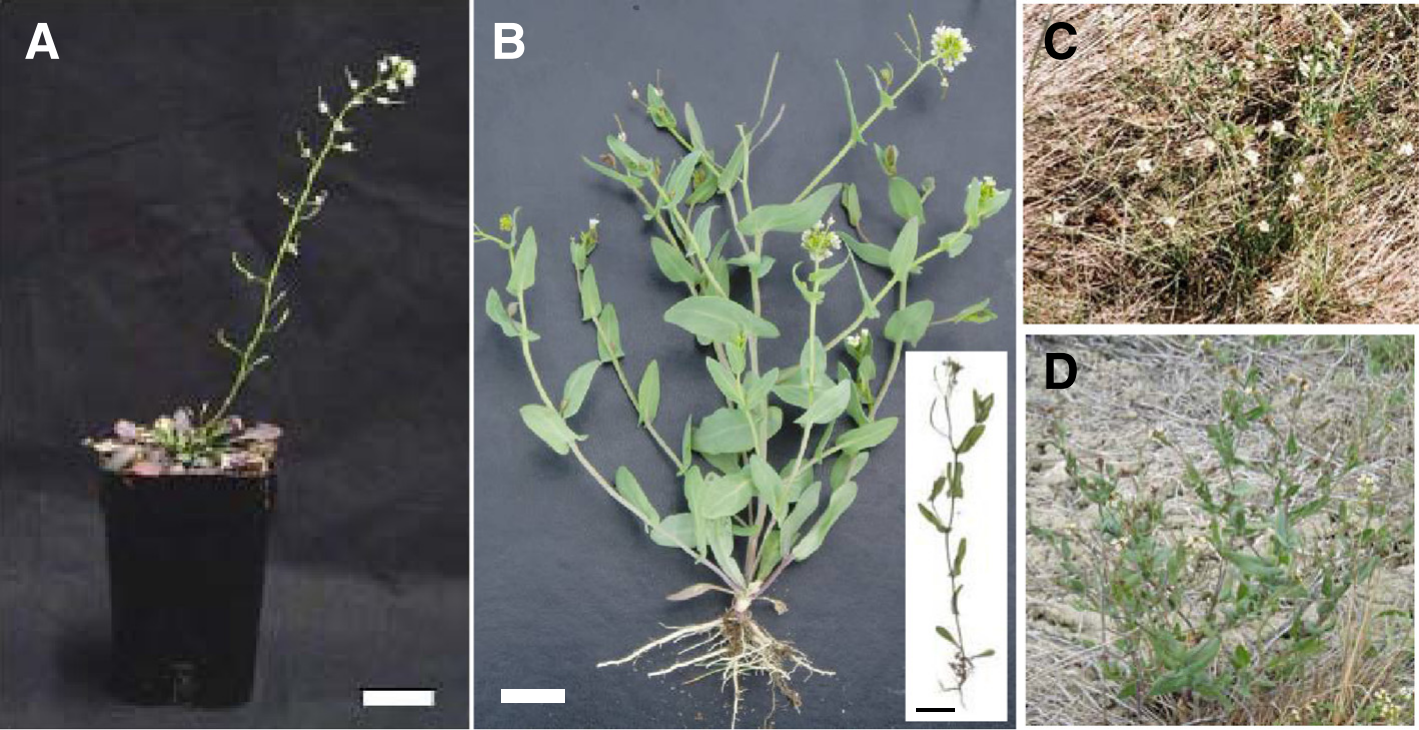
**Phenotype of *Thellungiella *grown in controlled environments and at the Yukon field site. A**) 9-week old *Thellungiella* grown in controlled environment cabinets showing seed-bearing siliques and a prominent rosette (scale: white or black bar =2 cm). **B**) *Thellungiella* found in the field has cauline leaves borne on multiple stems that terminate in flowers and siliques, but lack the prominent rosette observed in plants grown in growth cabinets. The inset shows an example of *Thellungiella* found in the field with one main stem. **C**) *Thellungiella* growing on Yukon field site in 2003 amongst dry vegetation. **D**) *Thellungiella* growing on Yukon field site in 2005.

The natural accession of *Thellungiella* found in the Yukon exhibits a different phenotype *in situ* than in cabinet growth conditions. In growth cabinets set to mimic the temperature range and summer day length of the Yukon, plants produced from seed collected from the same Yukon field site develop multiple (60 to 80 by 8 weeks) basal rosette leaves (Figure 1A) with flowers first appearing at about 4 to 5 weeks as a small cluster within the rosette. Plants usually bolt at about six weeks after germination and additional flowers are borne on a main stem. At about 8 weeks, axillary stems bearing flowers appear and occasional cauline leaves develop on the main and axillary stems with the basal rosette leaves remaining a prominent feature. In contrast, *Thellungiella* at field sites have prominent cauline leaves that clasp around the main and axillary flowering stems and either lack or have only a few, diminutive rosette leaves (Figure 1B, C, and D). Chemical analysis of leaf tissue shows N, Ca^++^, and Mg^++^ contents to be similar for cabinet and field-grown plants, but the Na^+^ content of leaves harvested in the field is over six-fold higher than that of the cabinet-grown control plants (Additional file 1: Table S1).

Plants collected in 2003 experienced below-average precipitation in a 60 d period before harvest. In the same timeframe, plants collected in 2005 experienced more frequent episodes of rainfall leading to above-average cumulative precipitation. The contrasting precipitation patterns allowed us to compare field plants exposed to water deficits in 2003 with those experiencing above-normal cumulative precipitation in 2005. Figure 1C shows the grass surrounding *Thellungiella* to be dead or dying in 2003. *Thellungiella* plants were small (8 to 18 cm tall) but green and either flowering or setting seed. In contrast, at the same site in 2005 the above-average precipitation patterns contributed to an abundance of green vegetation around the *Thellungiella* plants that were themselves almost 2- to 3-fold larger than the plants found in 2003 (Figure 1B and D).

### Profiling of *Thellungiella* plants grown under field conditions

To prepare transcript profiles of field plants we used a cDNA microarray containing 3628 unique sequences derived from libraries enriched in stress-responsive cDNAs as described in Wong et al. [19]. Field expression data was quantified relative to the signal intensity of control cDNA prepared from cauline leaves of plants grown in controlled environment chambers. Of the gene products represented on the microarray, 673 (19%) were differentially expressed within cauline leaves of *Thellungiella* growing in the field relative to cabinet-grown controls (Additional file 2). In 2003, transcripts associated with 216 cDNAs were differentially expressed with 132 and 84 being present at higher or lower levels, respectively, relative to cabinet-grown plants. For plants sampled in 2005, transcripts associated with 548 genes were differentially expressed with 301 and 247 being present at higher or lower levels, respectively, relative to plants grown in chambers. Of the 673 genes differentially expressed in field samples, only 7% were expressed at higher levels over chamber-grown controls in both years and 6% were expressed to a lesser extent in both field years.

**Figure 2:**
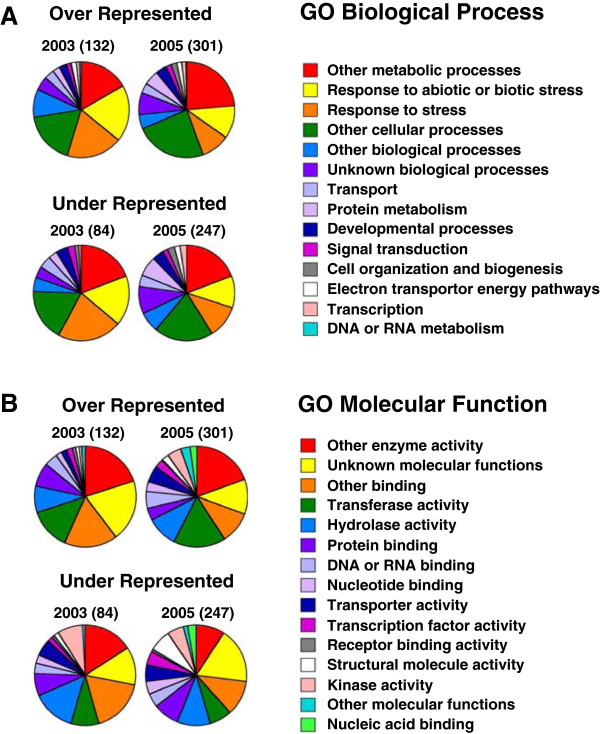
**GO classification of transcripts differentially expressed in *Thellungiella *harvested at a Yukon field site.** The genes whose transcripts were enhanced or repressed in Yukon *Thellungiella* harvested from the field in 2003 and 2005 relative to growth chamber grown plants were classified into the (**A**) biological processes and (**B**) molecular function categories using the TAIR9 GO categorization (http://www.arabidopsis.org/tools/bulk/go/index.jsp). Numbers in brackets are the total number of genes contributing to the pie chart.

Given the diversity of environmental conditions possible at this field site in the Yukon, it is reasonable to ask whether *Thellungiella* plants that have adapted to life in a harsh climate manifested a stress response during either year relative to our cabinet-grown controls. To address this question, we classified genes showing differential expression in 2003 or 2005 compared to cabinet controls by the biological processes and molecular functions encoded by their products according to the Gene Ontology (GO) annotations (http://www.arabidopsis.org) (Figure 2). The theoretical expectation for a proportion of genes classified by GO Biological Process for “response to stress or biotic stimulus” and “response to stress” given the coding potential of the Arabidopsis genome is 8.4% (TAIR; http://www.arabidopsis.org/tools/bulk/go/) and for unigenes represented on our microarray chip is 8% [14]. The GO Biological Process categorizes 180 of the 673 (~27%) genes showing differential expression in plants sourced from the field relative to chamber-grown plants as being associated with stress (Figure 2A; Additional file 2). However, gene products classified as stress-responsive comprised a two-fold greater proportion of the transcripts showing enhanced expression in 2003 (38%) compared to 2005 (19%), consistent with *Thellungiella* plants in 2003 being more stressed than plants in 2005 (Figure 2A). This interpretation of the functional gene classification is corroborated by size and physiological measurements of the field plants. Field-grown *Thellungiella* sampled in 2003 were of small stature (Figure 1C) and plants had a lower solute potential than either cabinet-grown plants or plants collected in 2005 indicating that they had accumulated solutes to a greater extent. Specifically, at approximately 9 weeks of age, a well-watered Yukon plant in the growth cabinet has a cauline leaf solute potential (ψ_S_) of -1.50 ± 0.05 MPa, a comparatively low value for an unstressed plant [27]. In comparison, visibly turgid cauline leaves from field plants were -2.07 ± 0.13 MPa and -1.63 ± 0.07 MPa in 2003 and 2005, respectively. Interestingly, GO classification by molecular function shows that genes encoding proteins of unknown function were expressed at a proportionately higher level in 2003 while the kinase class made little to no contribution (Figure 2B).

### Patterns among gene products of field-grown *Thellungiella* and cabinet-grown plants subjected to environmental stress

**Figure 3:**
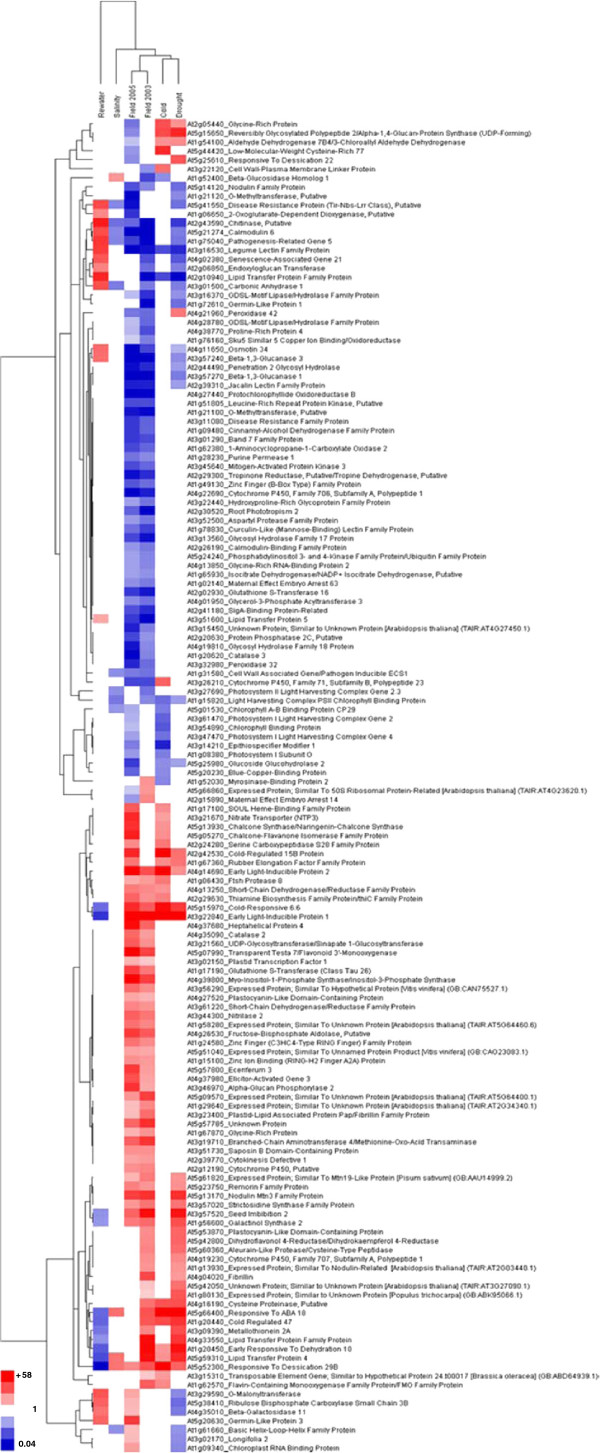
**HCA of genes differentially expressed in *Thellungiella *harvested from the Yukon field site in 2003 and 2005 and *Thellungiella *exposed to abiotic stresses in growth cabinets.** Transcript expression levels that were significantly different from control levels were converted to a ratio of the treatment over the appropriate control. Ratios greater than 1.5 or less than 0.67 were then log_10_ transformed before being subjected to HCA and illustrated as a heat map. White indicates no difference in gene expression between the sample and its respective control while red or blue indicates that gene expression was higher or lower, respectively, relative to a control. Values associated with the variable intensity of red and blue in the legend are fold-differences (before log transformation) compared to the appropriate control. Genes are annotated with the Arabidopsis locus identifier to which they are best matched.

The microarray dataset reported by Wong et al [19] for Yukon *Thellungiella* plants subjected to stress treatments under cabinet conditions was used in a meta-analysis for comparison with the expression profiles of field plants. Among the 673 genes described above, only 85 genes showed differential expression in the field and in response to at least one growth cabinet stress treatment. There were 63 genes differentially expressed in leaf samples collected in 2003 and 2005 that were not differentially expressed following any of the growth cabinet imposed stresses. This subset of 148 genes was subjected to hierarchical cluster analysis (HCA) to discern patterns of differential gene expression between field and cabinet stress treatments with the understanding that 2003 was a more stressful year. The dendrogram at the top of the HCA shown in Figure 3 groups the two gene expression datasets obtained from field plants together into a single group. The dataset that shows the greatest difference from the others is the one derived from re-watered plants recovering from a simulated drought stress. The top half of the figure includes genes whose transcripts were down-regulated (blue) in the field relative to control, cauline leaves of cabinet-grown plants while the bottom half includes genes found to be more highly expressed (red) in the field. The first six genes at the top of the figure show comparatively strong stress-responsive expression in plants from growth cabinets exposed to cold or drought treatments but they showed no increased abundance in cauline leaves of field plants relative to chamber-grown plants for either year sampled. Other genes show changes in expression that support a role in stress acclimation in both field and cabinet grown plants. For example, a group of seven genes at the base of the HCA heat map showed increased expression in 2005 and in leaves of plants re-watered following drought treatment in cabinets but showed no increase in expression in 2003 or following drought, cold or salinity treatments in cabinets. This group may be comprised of genes whose expression is down-regulated during stress or associated with plants growing under more favorable cabinet and field conditions such as those of 2005.

**Figure 4:**
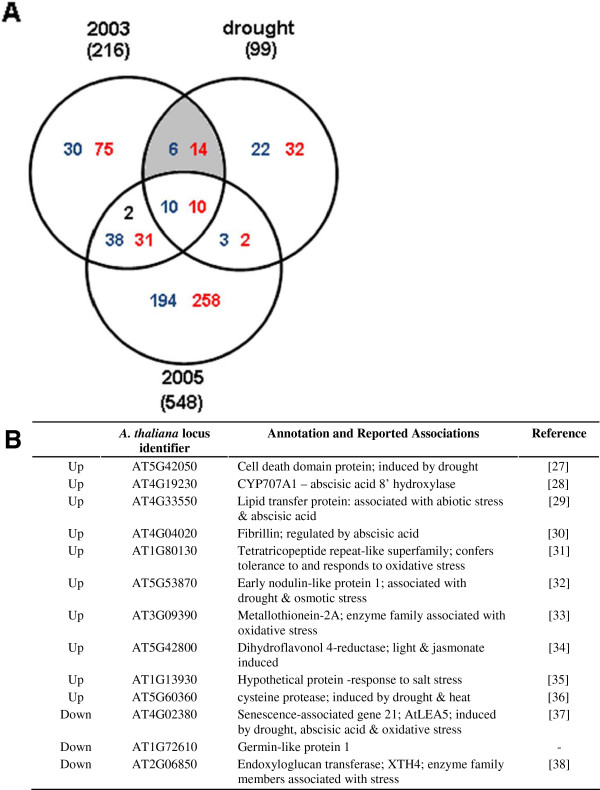
**Exploring drought-associated genes of *Thellungiella *grown at the field site and in the growth cabinet. A**) Relationships between genes differentially expressed in *Thellungiella* at the Yukon field site and plants drought-stressed in cabinets compared to cabinet controls are presented in Venn diagram form. The numbers of genes indicated in blue text are down-regulated relative to cabinet controls and the numbers of genes presented in red text are up-regulated relative to cabinet controls. Black text represents the number of genes regulated in opposite directions in 2003 compared to 2005. **B**) Summary list of candidate drought-responsive genes in *Thellungiella*. 20 genes (shaded region in panel **A**) showed differential expression in 2003 and in cabinet-grown plants subjected to simulated drought stress compared to cabinet controls. A subset of 13 genes whose expression was not influenced by either salt or cold exposure is presented [28-39].

We expect that genes whose products are critical for growth under a given stress condition will be expressed irrespective of whether the plants are grown and stressed in cabinets or in the field. This premise can be tested using the dataset for differentially expressed genes (Additional file 2). For example, genes responsive to water deficits are less likely to show differential expression in 2005 when rainfall was abundant. On the other hand, plants harvested in 2003 and chamber-grown plants exposed to a simulated drought should show overlap in drought-responsive genes. Figure 4A shows a Venn diagram of drought-responsive genes in the growth cabinet and their overlap, if any, with differentially expressed genes in 2003 or 2005. A number of interesting inferences can be drawn considering that 2003 was a year of low precipitation. Firstly, approximately 4.5-fold more differentially expressed genes were only detected in 2005 and given the ample rainfall, their expression is inconsistent with genes whose products are responsive to drought; their expression must reflect other field-related conditions in 2005. We found 99 genes showing drought-responsive behavior in cabinets although cold and salinity also influenced the expression of many of these genes. Only a subset of 20 genes (shaded overlap) showed differential expression in 2003 samples and in drought-treated plants in growth chambers. Of this group, 13 genes were not responsive to either salinity or cold and the direction of change was the same (10 up and 3 down-regulated relative to the gene expression of control plants; Figures 3 and 4A). The expression pattern of this sub-set of *Thellungiella* genes suggests that they are responsive to water deficits whether engineered under controlled conditions in a cabinet or naturally in the field. Of these 13 genes, the functions associated with several are unknown but a number have documented roles in osmotic stress (see references [28-39] cited in Figure 4B). The genes of unknown function deserve further study as their expression patterns implicate these products in processes required to tolerate water deficits under stressful field conditions.

### Metabolite profiling of *Thellungiella* plants grown under field conditions

The GO annotation analysis of Figure 2 shows that transcripts associated with metabolism comprise one of the largest categories of differentially regulated genes in *Thellungiella* field plants. In order to gain greater insight into the biochemical activities of these plants we extracted and profiled polar metabolites by gas chromatography/mass spectrometry (GC/MS). Using this approach, it was possible to simultaneously monitor over 300 mass spectral tags (MSTs) corresponding to chemically diverse compounds.

Plant metabolites can undergo diurnal changes in abundance [40]. With this consideration and the restricted access to the field site, it was important to identify which metabolites undergo diurnal changes in abundance and the time point that would provide the most comprehensive “snapshot” of chemical components. We sampled leaf tissue harvested at the field site at three time-points corresponding to 2, 7 and 12 h after sunrise. Measurements of MST abundance at any given time point were expressed as an average fold-change of the three time points selected for tissue analysis and the 29 MSTs found to undergo significant changes in abundance were analyzed by HCA (Additional file 1: Figure S2). The majority of the compounds that underwent changes in content showed maximum abundance in the samples obtained 7 h after sunrise. This group includes *myo*-inositol, raffinose, galactinol, quinic acid, fructose, sucrose, and 16 unidentified MSTs. Members of the raffinose family oligosaccharides have been implicated in desiccation and freezing tolerance of a variety of plants. While one might expect the content of protective compounds to remain stable in the face of a persistent osmotic stress, such as that found at a highly saline field site, diurnal changes have been observed for *myo*-inositol, fructose, and raffinose for unwatered coleus [41] and so diurnal changes in these components neither supports nor precludes a role for raffinose family oligosaccharides in osmotic adjustment of *Thellungiella*. A smaller group whose abundance was highest in early morning (2 h) samples included phosphate, citrate, glycine, and unknown 1320 while one MST, unknown 966, was most abundant in the late time-point sample (12 h after sunrise). Given the difficulty of sampling at a remote field site and the pattern of highest abundance tending to be near midday, we selected a single time-point corresponding to 7 h after sunrise for routine sampling and synchronized this sampling time for comparisons with plants in growth cabinet material. Our findings agree with those of Gibon et al. [40] who reported that metabolite abundance in Arabidopsis is relatively low early in the day and increases over the course of the day.

**Figure 5:**
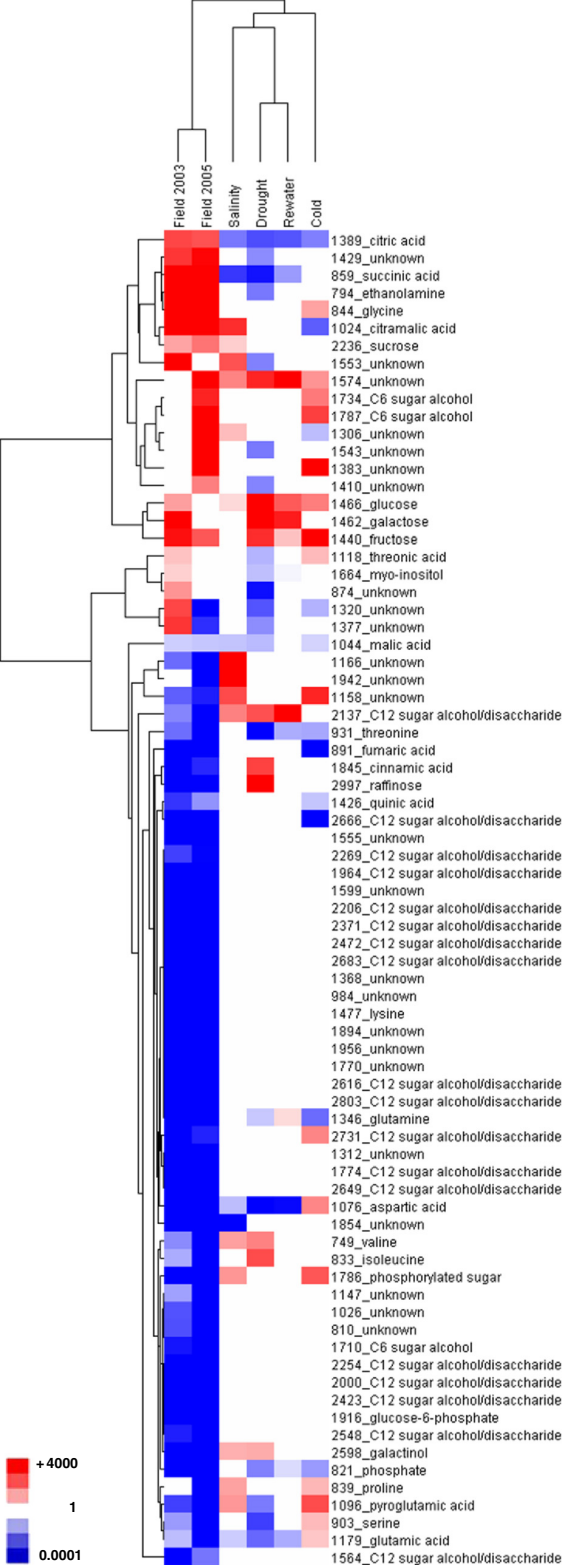
**HCA of metabolites in *Thellungiella *harvested from the Yukon field site in 2003 and 2005 and chamber grown plants exposed to abiotic stresses.** Illustrated is a heat-map of an HCA using 76 MSTs showing differential abundance between field and cabinet stress treatment plants over cabinet controls. For inclusion, a given MST was either found in profiles of leaf extracts for both field years or in the profiles of extracts in a single field year in cases where the same MST was found in profiles of stressed, cabinet-grown plants. The mean MST abundance was expressed as the fold ratio of treated or field over cabinet control levels. Those ratios that exceeded 1.5 or were lower than 0.67 were then log_10_ transformed before being subjected to HCA and illustrated as a heat map. Colors and legends as per Figure 3.

We found 109 MSTs in leaf extracts prepared from plants collected in 2003 and 2005 that showed statistically significant differences in abundance relative to MSTs present in extracts of cauline leaf samples from chamber-grown controls (Additional file 3). A subset of 76 MSTs that showed changes in abundance in field plants only, or between field and cabinet stress treatment samples, were subjected to HCA (Figure 5). A striking impression shown by this HCA is that most components, including 19 MSTs tentatively identified as sugar alcohols, were less abundant in leaves of field plants than cauline leaves from growth cabinet control plants. Leaves harvested from *Thellungiella* growing on saline field sites in 2003 and 2005 had a higher content of citrate, unknown 1429, succinate, ethanolamine, glycine, citramalate, sucrose, and fructose compared to cauline leaf controls. None of the laboratory stress conditions tested led to an increased relative abundance of the first four MSTs in this list. Among the low abundance metabolites in field samples were some found to be present at higher levels following at least one of the stress treatments in cabinets. This latter group includes cinnamic acid, raffinose, aspartate, valine, isoleucine, galactinol, proline, serine, and glutamate.

### Physiological adjustments to salt exposure include plasticity in organic solutes accumulated by Yukon *Thellungiella* plants

A general mechanism for tolerance to osmotic stress among plants involves the accumulation of organic solutes to serve various roles including scavenging free radicals, detoxification and osmotic adjustment [42]. There is ample experimental evidence that the Shandong ecotype of *Thellungiella* accumulates proline in response to NaCl treatments, a trait observed in numerous plant species, [17,20-22,43]. In contrast, we found little evidence that *Thellungiella* plants harvested at the Yukon field site contained proline at levels exceeding those found in rosette leaves of well-watered chamber-grown plants (Figure 5). This outcome was unexpected because our soil analysis and visual observations of the field location show that the plants are growing under saline conditions (Figure 1 and Additional file 1: Table S1). Tissue sampled from the field was enriched in Na^+^ compared to cabinet controls and leaf ψ_S_ values from field plants were lower than non-salinized plants in growth chambers. Furthermore, our HCA of differentially expressed genes between field and chamber-grown plants (Figure 3) does not implicate gene products associated with proline synthesis under stress such as the *Thellungiella* ortholog of Δ *1-pyrroline-5-carboxylate synthetase 1*[19,44].

**Figure 6:**
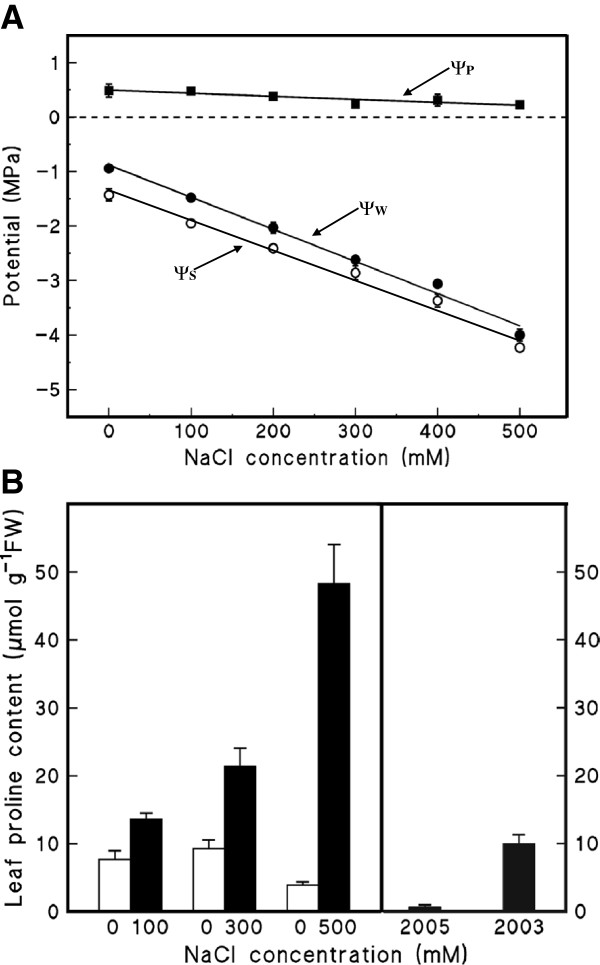
**Physiology of Yukon *Thellungiella *plants subjected to salt treatments. A**) Water relations of Yukon *Thellungiella* exposed to NaCl. Plants were salinized step-wise by increasing the concentration of NaCl in 50 mM increments. Leaf ψ_W_ and ψ_S_ measurements were taken after 3 d of acclimation to each salt concentration. The ψ_P_ was estimated as the difference between ψ_W_ and ψ_S_. Values are means of six individual plants ± SE. **B**) Leaf proline content of Yukon *Thellungiella* subjected to NaCl treatments or sampled at the field site in 2003 and 2005. Values are means ± SE from five individual plants.

We further explored the relationship between NaCl treatment of plants in cabinets and proline accumulation by comparing Yukon *Thellungiella* plants that were well watered with those that were salinized step-wise up to 500 mM NaCl. *Thellungiella* salinized in growth cabinets will flower and set seeds even when irrigated with 500 mM NaCl. The salinized plants showed a reduction in leaf ψ_W_ and ψ_S_ but remained turgid (Figure 6A) and the proline content of leaves underwent a salt-responsive increase (Figure 6B). Although this experiment confirmed the capacity for Yukon *Thellungiella* to accumulate proline in growth cabinets, it did not satisfactorily answer why we failed to observe proline accumulate to comparable levels in the field plants (Figure 5 and 6B). We hypothesized that field-related conditions influence the nature of solutes accumulated by Yukon *Thellungiella* plants, with one factor being the availability of nutrients. There is evidence that nitrogen can influence the organic solute composition of salt-stressed *Spartina alterniflora* and *Wollastonia biflora*[45,46].

### Nitrogen availability influences the array of solutes accumulated by Yukon *Thellungiella* during salt exposure

**Figure 7:**
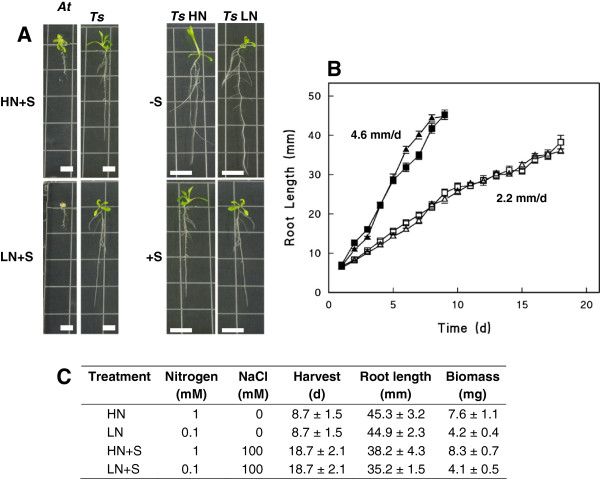
**Growth of *Thellungiella *seedlings exposed to nitrogen deficit and/or salt stress. A**) Representative *Thellungiella salsuginea* (*Ts)* and *Arabidopsis thaliana* (*At*) seedlings grown on media containing 1 mM or 0.1 mM nitrogen (HN and LN, respectively) in the presence (+S) or absence (-S) of 100 mM NaCl. Scale bars = 6.5 mm. **B**) Primary root elongation kinetics of *Thellungiella* seedlings. Treatments shown are: HN, ■; LN, ▴; HN + S, □; LN + S, ▵. Root measurements were taken at the same time daily. All data are presented as the mean ± SE of 40 seedlings per measurement across three biological replicates. Growth rates for roots of seedlings grown in the presence or absence of NaCl are presented in mm·d^-1^. **C**) The extent of *Thellungiella* shoot biomass and root length at harvest after growing on media containing varying concentrations of nitrogen and salt.

Given the low nitrogen content of soil at the field site (Additional file 1: Table S1), we reasoned that nitrogen availability could influence the production and accumulation of proline by plants in the field when compared to plants in the cabinets that were fertilized weekly. To address this question we grew seedlings on defined nutrient media and subjected them to one of four treatments: 1 mM nitrogen (HN), 0.1 mM nitrogen (LN), 1 mM nitrogen and 100 mM NaCl (HN + S), or 0.1 mM nitrogen and 100 mM NaCl (LN + S). Arabidopsis seedlings were stunted on HN + S and died on LN + S treatment plates. In contrast, *Thellungiella* seedlings exposed to 100 mM NaCl survived and grew under both HN and LN conditions (Figure 7A). *Thellungiella* growth was slower on media containing NaCl compared to media without added salt. The length of *Thellungiella* seedling primary roots was measured daily. *Thellungiella* roots grown in the absence of salt (LN and HN) grew at the same rate of 4.6 mm. d^-1^ while those exposed to 100 mM NaCl (LN + S and HN + S) grew at 2.2 mm·d^-1^, half the rate of the non-salinized seedling roots (Figure 7B). The nitrogen concentration of the media therefore did not affect primary root growth rate but the presence of added salt led to slower rates of root elongation. Shoot fresh weight measurements were taken on the day of harvest, defined as the day that primary roots reached the bottom of a vertically-oriented agar plate. The salinized roots grew more slowly and, accordingly, shoot tissue was collected from LN and HN seedlings at 9 d and from LN + S and HN + S seedlings at 19 d (Figure 7C). At harvest, shoot biomass was two-fold greater for seedlings grown on HN media compared to LN plates and this difference was not affected by salt (Figure 7C).

GC/MS was used to identify qualitative and quantitative changes in organic solutes extracted from harvested shoots. We identified 49 MSTs as showing significantly different relative abundance between the four treatments; 12 were identified as probable sugars based upon mass spectral information but 23 could not be associated with any chemical class (Additional file 4).

**Figure 8:**
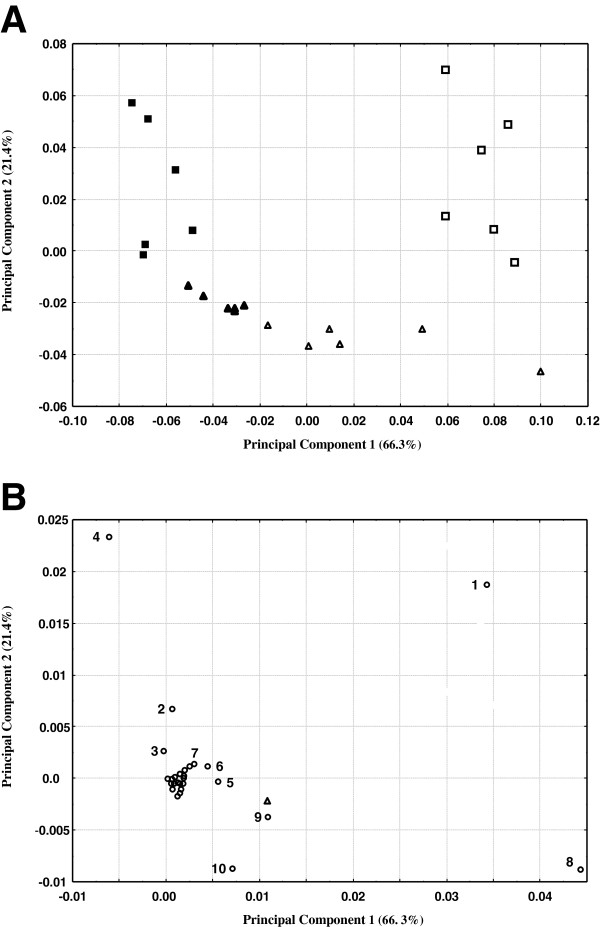
**Principal component analysis of 49 MSTs differing in relative abundance following plant exposure to HN, LN, HN + S, or LN + S conditions. A**) Sample measurements for each treatment consisted of 3 biological replicates of extracts from pooled seedling shoot tissue analyzed in duplicate. Each point represents the dataset comprised of 49 MSTs in these six analyses and is positioned on a biplot of principal component 1 vs. 2. (■ = HN, □ = HN + S, ▴ = LN, **▵** = LN + S. **B**) Projections of metabolite variables onto the principal components described in A. Numbered metabolites are: 1 = proline, 2 = serine, 3 = threonine, 4 = pyroglutamic acid, 5 = unknown 1429, 6 = fructose, 7 = sugar 1579, 8 = *myo*-inositol, 9 = sugar 2100 and 10 = raffinose.

Abundance estimates for the 49 MSTs across the four treatments were subjected to principal component analysis (PCA). PCA simplifies the large volume of data (all MSTs from the multiple GC/MS analyses) and helps reveal sources of major variation in MST abundance [47]. PC1 (principal component 1) and PC2 together account for 87% of the variance in the observations corresponding to the four treatments tested, with PC1 accounting for 66.3% (Figure 8A).

On this PCA, observations obtained from individual plants exposed to the same test media group together (Figure 8A), suggesting that variance between treatments was greater than variance within treatments. Observations derived from seedlings grown in the absence of salt (LN and HN) and those from plants grown in the presence of salt (LN + S, HN + S) are discriminated along the PC1 axis, indicating that PC1 is primarily associated with variance in metabolites due to seedling salt exposure. Observations derived from the LN and LN + S are separated from HN and HN + S treatments along the PC2 axis, indicating that PC2 is associated with variance due to seedling exposure to nitrogen (Figure 8A).

The PCA data was analyzed further to identify metabolite variables that influence the position of the observations along PC1 and PC2. Projections of metabolite variables onto the two principal components yielded values near the origin for the majority of metabolites (Figure 8B). However, ten of the 49 variables were clearly separable from the rest and these included fructose, *myo*-inositol, sugar 2100 and raffinose. These particular variables lie below zero on the PC2 axis and contribute towards the position of the LN + S observations. Points corresponding to the variables pyroglutamic acid and serine are found on the left side of the PC1 axis and above zero on the PC2 axis where they contribute to the position of the HN observations. In contrast, proline has a strong positive loading for both PC1 and PC2 axes and so contributes to the position of the HN + S observations. These results show that a change in abundance among comparatively few MSTs strongly influenced the position and separation of observations produced following PCA of MST data from the *Thellungiella* seedlings used in this study. Furthermore, much of the variance in this dataset is due to seedling proline content. Additional file 4 shows that the 45-fold increase in proline abundance between HN and HN + S plants can be contrasted with the fold-increases for several sugars between LN and LN + S plants (including raffinose, *myo*-inositol, fructose, sugars 2100 and 1579). This observation is consistent with our hypothesis that carbohydrates replace proline when plants are salt-stressed under nitrogen limiting conditions.

## Conclusions

Even for a plant that grows in a high stress environment, there was considerable plasticity in variance to abiotic stressors. Predictably, in our comparisons between field- and cabinet-grown *Thellungiella* plants we found many differences in gene expression and metabolite content. However, there were consistent patterns among stress-responsive transcripts and metabolites that strongly associate a subset of gene products with a role in the adaptive capacity of this plant to survive on saline soils and under drought conditions. The converse is also true. One trait that has a long association with stress tolerance, including salinity tolerance of *Thellungiella*[20,21,43], is that of proline accumulation. Surprisingly, we found no evidence for elevated proline content in leaves of plants that grew in salt-laden Yukon soils despite the fact that plants descended from seeds of the same population grown in cabinets accumulate this amino acid in a stress-responsive manner. In this report we show evidence that for at least this trait, the availability of nutrients influences the array of organic solutes accumulated by *Thellungiella* growing under saline conditions. We propose that this plasticity is not an exclusive property of proline metabolism but a trait that reflects a more fundamental flexibility required for *Thellungiella*’s extremophile lifestyle. As such, we hypothesize that stress-responsive traits in common between field and growth cabinet locations is not a serendipitous overlap but likely reflects their essential requirement for stress tolerance. Comparisons between field and laboratory grown plants offer a promising approach to revealing candidate gene leads for improved crop performance under adverse environmental conditions.

## Methods

### *Thellungiella* at Yukon field sites

In 2002, 2003, and 2005 cauline leaf tissue was harvested from mature, flowering Yukon *Thellungiella* at a field site near Whitehorse, Yukon (location: 60° 55.928′N, 135° 10.249′ W; elevation 647 m). Leaf tissue from individual plants was transferred quickly to 2 mL Nalgene cryovials, immediately flash frozen in liquid nitrogen, and then transferred to a charged MVE XC20/3V vapour shipper (Jencons Scientific Inc, Bridgeville, PA) where samples were kept frozen at -150°C for transport. Vials were transferred to -80°C for storage pending analysis.

### Controlled environment plant growth conditions and stress treatments

Seeds of the Yukon genotype that had been sourced from the field site and bulked at McMaster University were sterilized using a vapour-phase gas technique [48] and then mixed with 0.1% (w/v) Phytagel (Sigma) and applied by pipette onto a moistened soil mixture containing six parts Promix BX (Premier Horticulture, Rivière-du-Loup, PQ) and one part Turface (Profile Products LLC, Buffalo, NY) in individual 5 × 5 × 7 cm pots. Seeds in pots were stratified for 2 d at 4°C before transfer to growth chambers (AC 60 Enconair, Winnipeg, MB) set with a 21 h day and irradiance of 250 μmol m^-2^s^-1^ and 22°C/10°C day/night temperature regime. Plants were watered daily as needed and fertilized one time per week with 1 g L^-1^ 20-20-20 (N-P-K) fertilizer. For salinity treatments, four-week-old plants were watered with 50 mM NaCl for 3 d and the salinity level of the irrigating solution was increased in increments of 50 mM NaCl every 3 d to final concentrations of 100 mM, 300 mM, and 500 mM NaCl. Plants were watered for 3 d at their final salinity level prior to physiological analysis and harvest. For comparative determinations, treated plants were paired with age-matched unsalinized controls.

Yukon *Thellungiella* plants grown in controlled environment chambers as described above produce cauline leaves at approximately 8 weeks. Thus, cauline leaves collected from 12 week old cabinet-grown plants served as the source of unstressed tissue in microarray and metabolite profiling experiments involving cauline leaves from field-grown plants. Rosette leaves of chamber-grown plants served as the source of tissue for metabolite profiles comparing healthy, cold, drought, drought/re-watered, and salinized plant samples. Tissue from individual plants (un-pooled) was used for all analyses.

For seedling nutrient stress experiments, *Thellungiella* or Arabidopsis (Col-0) seeds were sterilized and stratified as above on MS agar plates. Four days after germination, seedlings were transferred to solid media plates composed of Hoagland's nutrient solution [49] in which the amount of nitrogen (nitrate) was 0.1 or 1 mM and salt (NaCl) was 0 or 100 mM [50]. Treatment plates containing seedlings were incubated vertically in a growth chamber with a 12 h photoperiod set at 22°C and 150-200 μmol m^-2^s^-1^ photon flux density. For metabolite analysis, shoots were harvested at the same root length stage of development across treatments. Tissue was pooled in 50 mg aliquots, flash frozen in liquid nitrogen and stored at -80°C until further use.

### Soil and physiological analyses

Soil samples were collected to a depth of 18 cm at the site where Yukon *Thellungiella* tissue was harvested. Soil pH, electrical conductivity, and chemical content were determined and the elemental composition of soil samples, field and chamber leaf tissues were analyzed [51].

Leaf water (ψ_W_) and solute (ψ_S_) potential measurements were performed using a HR33T psychrometer fitted with a C52 chamber (Wescor Inc., Logan, UT) using a 6 mm diameter disc excised from a mature fully expanded leaf [19]. Leaf turgor (ψ_p_) pressure was estimated as the difference between the water and solute potential measurements [52].

### Differential gene expression

Due to the scarcity of field-sourced material, total RNA extraction and microarray hybridization conditions were as reported by Wong et al. [19] except the mRNA was first amplified using the Ambion MessageAmp aRNA Amplification kit (Applied Biosystems) and the cDNA was primed with a random primer mix (Invitrogen). A single round of RNA amplification was carried out according to the manufacturer’s protocol using 1 μg of total RNA. To generate the probe for microarray hybridization, 40 μg of amplified RNA was reverse-transcribed to synthesize aminoallyl-labeled cDNA followed by coupling of the aminoallyl groups to either Cyanine 3 or 5 (Cy3/Cy5). The cDNA microarrays were hybridized with Cy3- and Cy5-labeled probe pairs prepared using RNA extracted from untreated cauline leaves of chamber grown *Thellungiella* and compared with similarly labeled products from cauline leaves of *Thellungiella* harvested at the Yukon field site.

Three biological replicates with dye swap as a technical replicate were used for leaf tissue harvested from the field in 2003 and 2005 and statistical analysis was therefore based on a total of six replicates per field year. ScanArray confocal scanning system and QuantArray data acquisition software (Perkin-Elmer) were used to obtain data, and after normalization, differentially expressed genes between growth chamber and field-grown Yukon *Thellungiella* were detected using a Bayesian model as previously described [19].

### Metabolite analysis

Polar metabolites for profiling by GC/MS were obtained from 200 mg of frozen *Thellungiella* tissue taken from an individual leaf or pooled seedling shoot tissue to which 50 μL of ribitol (2 mg mL^-1^) was added. Tissue was ground together with the ribitol standard to a fine powder in liquid nitrogen. Metabolite extraction using methanol and chloroform was as described by Fiehn et al. [47] and the aqueous phase containing polar metabolites was retained for this study. The retention times were converted to retention indices by adding a mixture of fatty acid standards [53]. Methoxymation and derivatization were as reported by Roessner et al. [53].

Samples were analyzed using a Trace DSQ GC-MS system (Thermo Finnigan) operated in the positive ion electron impact (EI^+^) full scan mode. Samples were diluted 25-fold with hexane and 1 μL was injected using a MPS 2 autosampler (Gerstel GmbH & Co., Mϋlheim, Germany). Chromatography was performed on a 30 m long, 0.25 mm ID and 0.25 μm film thickness Restek Rtx-5MS integra column (crossbond 5% diphenyl – 95% dimethyl polysiloxane; Chromatographic Specialties Inc., Brockville, ON) fused with a 10 m guard column of the same composition. The injection temperature was 230°C, and the ion source was kept at 200°C. The carrier gas was helium (>99.999% purity; VitalAire, Hamilton, ON) delivered at a constant flow rate of 1 mL min^-1^. The temperature program was set at 50°C for 2.5 min then temperature was first increased at a rate of 7.5°C min^-1^ to 70°C followed by 5°C min^-1^ ramp to a final temperature of 310°C where it remained for 6 min. Mass spectra were recorded at three scans per second with a scanning range of 50 to 650 m/z.

Automated mass spectral deconvolution and identification system (AMDIS) software was used to extract peak abundance and mass spectra information for each trimethylsilyl derivative resolved in GC/MS chromatograms [54]. This information was imported in tab-delimited format to the GC/MS Analysis Software Package (GASP) (available at: http://www.flintbox.com, [55]) where the retention index (RI) was calculated as described by Roessner et al. [53] and the relative abundance for each component was adjusted to reflect the estimated recovery of the internal standard ribitol. The term MST refers to an individual trimethylsilyl derivative identified by a characteristic RI and MS [56]. Identical MSTs from multiple GC/MS analyses were aligned with GASP and subjected to statistical tests to identify those associated with specific samples and/or treatments. An arbitrary threshold detection limit of 0.00005 for relative response factors associated with MSTs was used in determining abundance and fold-differences among chemical components from different samples.

The chemical identity of an MST was determined by comparing its RI and MS parameters to those obtained for authentic standards analyzed using the same instrument and experimental conditions. MSTs that shared m/z ratios with authentic standards that had different RIs were classified according to predicted chemical classes such as sugars or sugar phosphates. When necessary for confirmation of MST identities, chemical standards were co-injected with samples prepared from leaves or seedlings. Leaf proline content was determined by comparing sample relative response factor values for proline to those calculated from running eight known amounts of proline on the GC-MS.

### Statistical analysis

Transcripts found to be differentially expressed in the field relative to the cauline leaves of cabinet-grown plants were compared to those showing stress-related changes in abundance reported previously by Wong et al. [19]. The Wong et al. [19] dataset is comprised of 147 transcripts showing statistically significant stress-responsive changes in leaves of Yukon *Thellungiella* plants grown in cabinets and subjected to cold, drought, salinity, and drought followed by re-watering treatments. Transcripts showing a 1.5-fold greater or lesser difference in expression in *Thellungiella* harvested from the field compared to cabinet-grown controls or stress treated versus control cabinet-grown plants (*P* < 0.01) were converted to a ratio of the control, log_10_ transformed, then subjected to HCA. MSTs identified in metabolite profiling whose abundance was significantly different by ANOVA (*P* < 0.05) between treated and control or field and control plants were expressed as a ratio of the control, and were then log_10_ transformed before being subjected to HCA. A minimum of five plants were used from each source of tissue.

Euclidean distance was used to calculate the distance matrix and a complete linkage method was used for hierarchical clustering of MSTs and transcripts using the program Cluster [57]. Heat maps were constructed using the Java Treeview program (http://jtreeview.sourceforge.net, [58]).

For seedling analyses, GC-MS profiles were generated from three biological replicate experiments testing the four combinations of nitrogen and salt. Extracts for analysis by GC-MS were prepared from duplicate, seedling shoot samples pooled from each treatment. Handling of data with respect to peak identification was as described above. The aligned data of all three trials was subjected to ANOVA and only those MSTs that were statistically significant (*P* < 0.05) were related to the mean value for all four treatments. This relative value was then log _10_ transformed before being subjected to PCA using a covariance matrix.

## Abbreviations

GO: Gene ontology; HN: High nitrogen; LN: Low nitrogen; HCA: Hierarchical cluster analysis; MST: Mass spectral tags; PCA: Principal component analysis; S: Salt; SE: Standard error of the mean.

## Competing interests

The authors declare that they have no competing interests.

## Authors’ contributions

DRG, JD, and AT conducted research, provided experimental material, and compiled data that were reported and discussed in their graduate theses that are now included in this paper. MJC, EAW and PSS compiled data, wrote the primary draft, and edited the final draft for submission based upon input of co-authors. CEW, YL, AL, and BAM performed the microarray analysis using a cDNA chip they produced. CLP provided soil and plant elemental analyses and interpretation. DRG, AT, PSS, YW, BEM, PN, GBG, and EAW were involved in the metabolite profiling and data analysis. All authors read and approved the final manuscript.

## Supplementary Material

Additional file 1 **Table S1.** Chemical analysis of Yukon soil and Yukon*Thellungiella* plants from field site and controlled environment chambers. **Figure S1.** Meteorological conditions near Yukon field site. Data were taken from Environment Canada for the Whitehorse site for the months of May to July in 2003 and 2005 (http://www.climate.weatheroffice.ec.gc.ca/climate_normals/indexe.html). Asterisks denote dates on which tissue was harvested from *Thellungiella* plants growing in the field. **Figure S2.** Analysis of metabolites showing differential abundance in field plants over the course of a day. The mean of measurements from five individual plants harvested from the Yukon field site at 2 h, 7 h and 12 h from sun-rise were expressed as a ratio relative to the mean abundance for all three time-points. The fold ratios were then log_10_ transformed, subjected to HCA and presented as a heat map. MSTs whose levels were significantly different (*P* < 0.05) at any time-point from the average of the day are illustrated. White indicates no difference between the mean MST abundance at a given time point and the daily average while red or blue indicates that the MST was present at significantly higher or lower levels, respectively, compared to the daily average. The values in the legend are fold differences (before log transformation) compared to the daily average. Click here for file

Additional file 2 **Relative abundance of differentially regulated transcripts in field/cabinet plants.** Transcripts whose expression levels in 2003 and 2005 field-grown plants were significantly higher or lower than their expression in cabinet-grown control plants. Ratios of expression in the field plants over expression in the control plants are given. Click here for file

Additional file 3 **Relative abundance of metabolites in field/cabinet plant tissues.** MSTs whose levels in 2003 and 2005 field-grown plants were significantly higher or lower than in cabinet-grown control plants are listed. Ratios of the level in the field plants over the level in the control plants are given. Click here for file

Additional file 4 **Relative abundance of metabolites in nitrogen/salt experiments with seedlings.** 49 MSTs exhibited significant changes in abundance between seedlings grown in HN, LN, HN + S and LN + S conditions. These metabolites had statistically significant (*P* < 0.05) changes in their abundance compared to the mean metabolite levels of all four treatments. Values are expressed as average relative response factors ± SE and n = 6 for each condition. Click here for file
