# Supplementary material for: Transcriptomic and metabolomic analysis of Yukon Thellungiella plants grown in cabinets and their natural habitat show phenotypic plasticity
Source: BMC Plant Biol. 2012 Oct 1;12:175. doi: 10.1186/1471-2229-12-175 (PMC3568734; doi:10.1186/1471-2229-12-175)
Supplement: Additional file 1 — Table S1. Chemical analysis of Yukon soil and YukonThellungiella plants from field site and controlled environment chambers. Figure S1. Meteorological conditions near Yukon field site. Data were taken from Environment Canada for the Whitehorse site for the months of May to July in 2003 and 2005 (http://www.climate.weatheroffice.ec.gc.ca/climate_normals/indexe.html). Asterisks denote dates on which tissue was harvested from Thellungiella plants growing in the field. Figure S2. Analysis of metabolites showing differential abundance in field plants over the course of a day. The mean of measurements from five individual plants harvested from the Yukon field site at 2 h, 7 h and 12 h from sun-rise were expressed as a ratio relative to the mean abundance for all three time-points. The fold ratios were then log10 transformed, subjected to HCA and presented as a heat map. MSTs whose levels were significantly different (P < 0.05) at any time-point from the average of the day are illustrated. White indicates no difference between the mean MST abundance at a given time point and the daily average while red or blue indicates that the MST was present at significantly higher or lower levels, respectively, compared to the daily average. The values in the legend are fold differences (before log transformation) compared to the daily average. [file 1471-2229-12-175-S1.pdf]

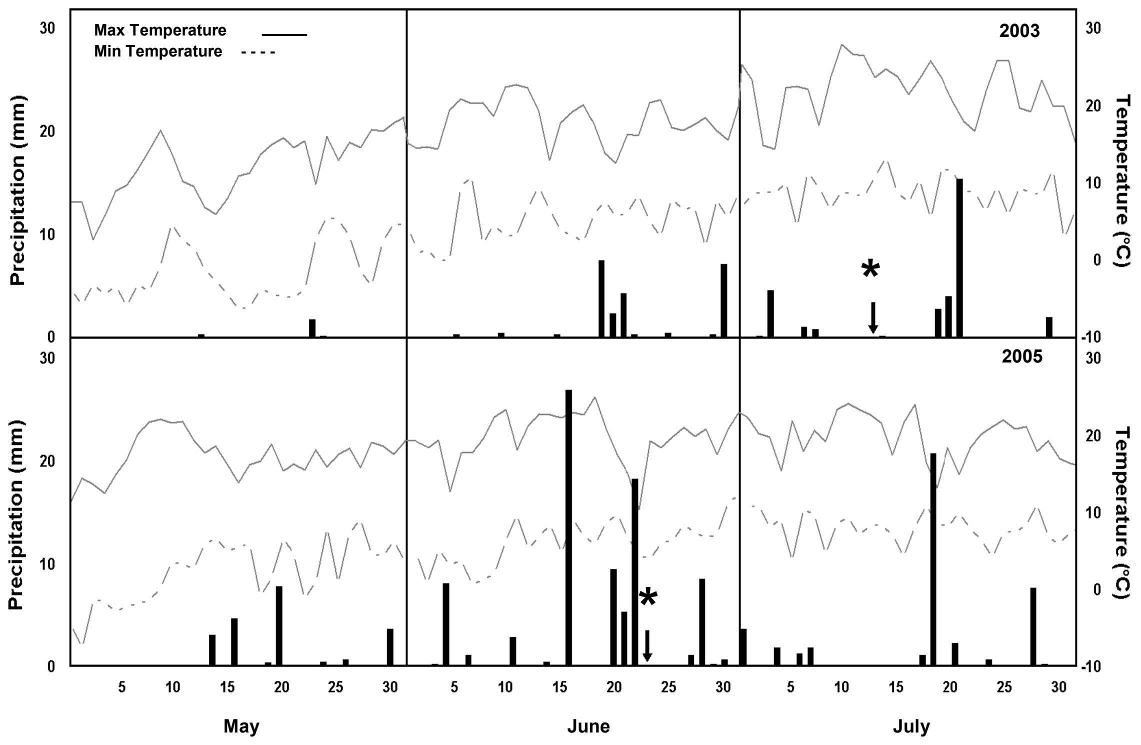

Additional File 1: Supplemental Figure S1. Meteorological conditions near Yukon field site

Data were taken from Environment Canada for the Whitehorse site and the months of May to July for 2003 and 2005 (http://www.climate.weatheroffice.ec.gc.ca/climate_normals/index e.html). Asterisks denote dates on which tissue was harvested from *Thellungiella* plants growing in the field.

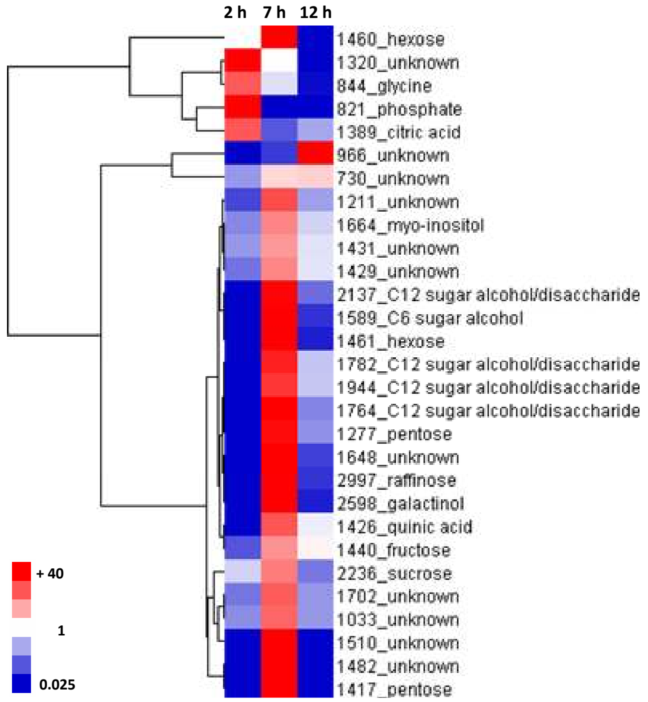

Additional File 1: Supplemental Figure S2. Analysis of metabolites showing differential abundance in field plants over the course of a day. The mean of measurements from five individual plants harvested from the Yukon field site at 2 h, 7 h and 12 h from sun-rise were expressed as a ratio relative to the mean abundance for all three time-points. The fold ratios were then $\log_{10}$ transformed, subjected to HCA and presented as a heat map. MSTs whose levels were significantly different ($P<0.05$) at any time-point from the average of the day are illustrated. White indicates no difference between the mean MST abundance at a given time point and the daily average while red or blue indicates that the MST is present at significantly higher or lower levels, respectively, compared to the daily average. The values in the legend are fold differences compared to the daily average.

Additional File 1: Supplemental Table S1. Chemical analysis of Yukon soil and Yukon *Thellungiella* plants from field site and controlled environment chambers

	Soil[a]	*Thellungiella*[b]	
	Field Site	Field Site	Chamber
pH	8.3		
E.C. (dS m^{-1})	15.68		
Total C (%)	2.61[c]		
Total N (%)	0.26[c]	3.19	4.20
Total S (%)		1.92	1.71
**Ions (ppm)**			
NH_4^+	1[c]		
NO_3^-	10[c]		
P	26[c]	2800	9200
SO_4^{2-}	9379		
K	236	22300	43700
Ca	521	15800	10400
Na	2224	13630	2058
Mg	1456	6000	4200
Fe		128	58
Cu		5.6	19.4
Zn		26	86
Mn		34	86
B		40	40
Cl	184		

[a] soil paste unless otherwise specified
[b] mineral content based upon dry matter
[c] measurement on air dry soil as opposed to paste extract
